# Supplementary material for: Arabidopsis PII Proteins Form Characteristic Foci in Chloroplasts Indicating Novel Properties in Protein Interaction and Degradation
Source: Int J Mol Sci. 2021 Nov 23;22(23):12666. doi: 10.3390/ijms222312666 (PMC8657445; doi:10.3390/ijms222312666)
Supplement: Supplementary file 1 [file ijms-22-12666-s001.zip › ijms-1474213-supplementary.pdf]

International Journal of
Molecular Sciences

MDPI

Supplementary Materials: *Arabidopsis* PII Proteins Form Characteristic Foci in Chloroplasts Indicating Novel Properties in Protein Interaction and Degradation

Natalie Krieger, Kai-Florian Pastryk, Karl Forchhammer and Üner Kolukisaoglu

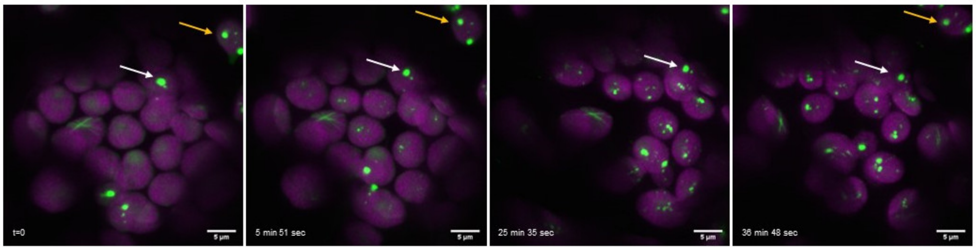

Figure S1. PII foci can be stable for more than 30 min. *At*PII-GFP (green) under control of the *p35S* promotor was expressed in transiently transformed *N. benthamiana* and aggregates in PII foci in chloroplasts (magenta). Z-Stacks of time series were acquired 3 days after transient transformation of *N. benthamiana* leaves. Arrows indicate PII foci found over the whole recorded time. Scale bar: 5 µm.

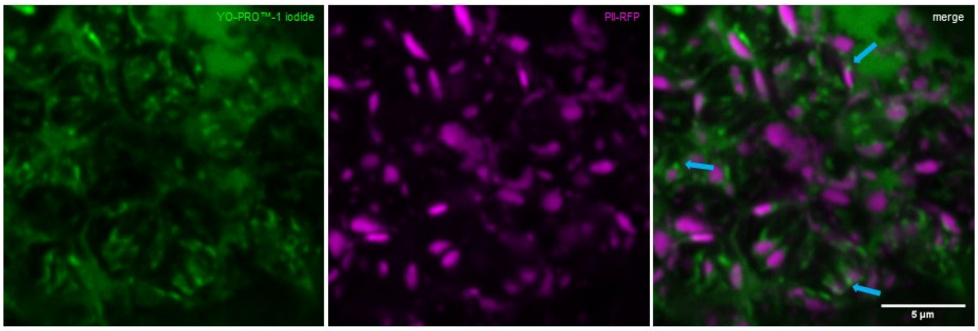

Figure S2. PII foci are not nucleoids. YO-PRO™-1 iodide stained nucleoids (green) in transiently transformed *N. benthamiana* leaves expressing *At*PII-RFP (magenta) under the control of the *p35S* promotor. *N. benthamiana* leaf disks were fixed overnight 3 days after transient transformation followed by staining with YO-PRO™-1 iodide for 15 min. Blue arrows indicate examples of PII foci proximal to nucleoids. Scale bar: 5 µm.

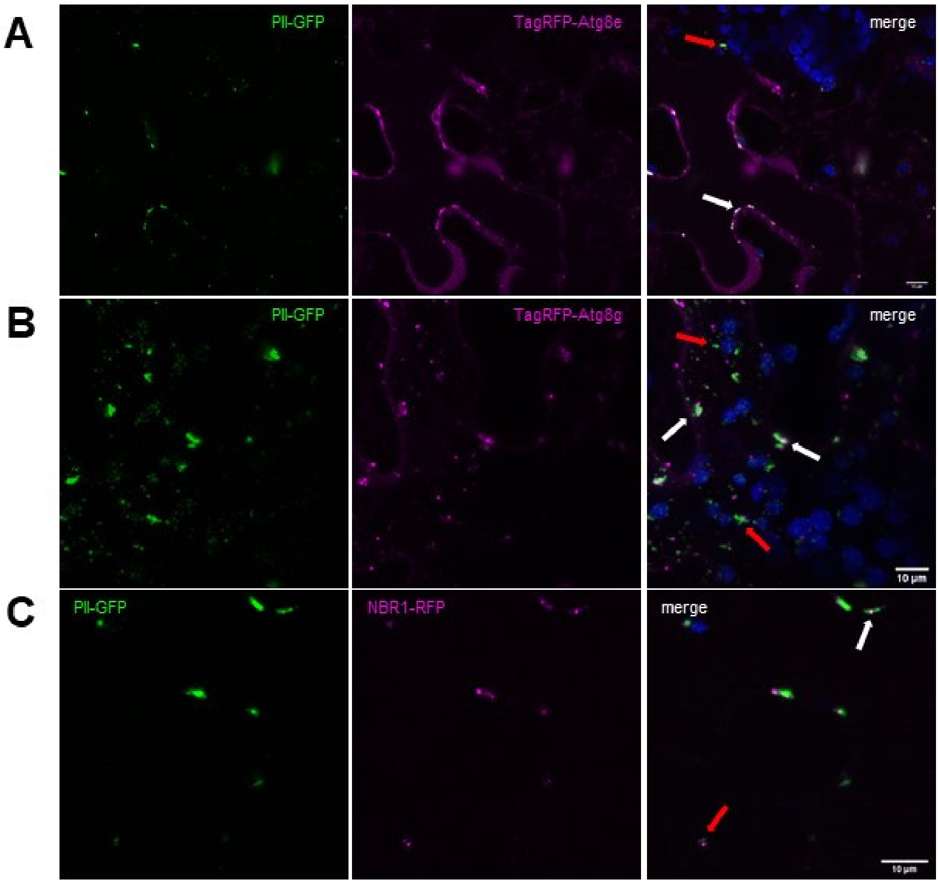

Figure S3. PII co-localizes partially with autophagy-related proteins. A)-C) TagRFP-*At*g8e (A), TagRFP-*At*g8g (B), and *At*NBR1-RFP (C), which all localize in the cytoplasm of *N. benthamiana*, were co-expressed with *At*PII-GFP (all genes under control of *p35S)*. Images were taken 2 days after transient transformation in *N. benthamiana*. GFP (green), TagRFP and RFP (magenta), brightfield (grey), autofluorescence (blue). White arrows: co-localization; red arrows: no co-localization. Scale bar= 10 µm.

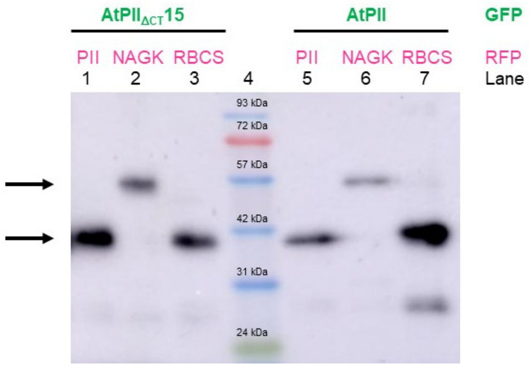

Figure S4. *At*PII$_{\Delta CT15}$-GFP can also bind to its interactors. *N. benthamiana* leaves were transiently co-transformed with *At*PII-GFP or *At*PII$_{\Delta CT15}$-GFP together with *At*PII-mCherry, *At*NAGK-mCherry or *At*RBCS3B- mCherry, respectively (all transformed constructs under the control of the *p35S* promotor). After 3 days of transient transformation GFP-fused proteins were immunopurified from protein extracts of transfected leaves. After SDS-PAGE of the Nano-Trap© eluates western blots were treated with anti-RFP antibodies to detect co-purified mCherry-fused proteins. In lanes 1-3 the eluates of extracts from co-expression of *At*PII$_{\Delta CT15}$-GFP with *At*PII-mCherry, **At**NAGK-mCherry or *At*RBCS3B-mCherry, respectively, have been loaded. In lanes 5-7 the same has been

done with extracts from co-expressions with *At*PII-GFP. The upper arrow indicates the predicted size for AtNAGK-mCherry (56.6 kDa) and the lower arrow the predicted sizes for *At*PII-mCherry and *At*RBCS3B-mCherry (40.2 kDa and 40.9 kDa, respectively).

Table S1. Primers used for the construction of the different plant expression vectors.

AtGLB1-Start	5′-caccATGGCGGCGTCAATGACGAAAC-3′
AtGLB1-End	5′-AGACGGTGAAAGCATATCACCAG-3′
AtPII-C2A	5′-ACGCTCACCTGTCCTAACTC-3′
NK_proAtPIIstart	5′-caccTTTTGTTTCACCTTAACCAG-3′
NK_AtNAGKstart	5′-caccATGGCCACCGTCACATCCAATGCTTC-3′
NK_AtNAGKend	5′-TCCAGTAATCATAGTTCCAGCTCCTTC-3′
NK_AtBCCP1start-2	5′-caccATGGCGTCTTCGTCGTTCTCAGTCAC-3′
NK-BCCP1end	5′-CGGTTGAACCACAAACAGAGGAGTGTC-3′
NK_RGCS1A-FP	5′-caccATGGCTTCCTCTATGCTCTCTTCCG-3′
NK_RGCS1A-RP	5′-ACCGGTGAAGCTTGGTGGCTTGTAGG-3′
NK_attP2P3-PIIstart	5′-GGGGACAACTTTGTATAATAAAGTTGTAATGGCGGCGTCAATGACG-3′
NK_attP2P3-PIIend	5′-GGGGACCACTTTGTACAAGAAAGCTGGGTTAGACGGTGAAAGCATATC-3′
NK_attP1P4-PIIstart	5′-GGGGACAAGTTTGTACAAAAAAGCAGGCTTAATGGCGGCGTCAATGACG-3′
NK_attP1P4-PIIend	5′-GGGGACAACTTTGTATAGAAAAGTTGGGTGAGACGGTGAAAGCATATC-3′
NK_attP1P4-NAGKstart	5′-GGGGACAAGTTTGTACAAAAAAGCAGGCTTAATGGCCACCGTCACATCC-3′
NK_attP1P4-NAGKend	5′-GGGGACAACTTTTGTATAGAAAAGTTGGGTGTCCAGTAATCATAGTTCC-3′
NK_attP1P4-BCCP1start	5′-GGGGACAAGTTTGTACAAAAAAGCAGGCTTAATGGCGTCTTCGTCGTTC-3′
NK_attP1P4-BCCP1end	5′-GGGGACAACTTTGTATAGAAAAGTTGGGTGCGGTTGAACCACAAACAG-3′
NK_RGCS1A-P1P4-FP	5′-GGGGACAAGTTTGTACAAAAAAGCAGGCTTAATGGCTTCCTCTATGCTC-3′
NK_RGCS1A-P1P4-RP	5′-GGGGACAACTTTGTATAGAAAAGTTGGGTGACCGGTGAAGCTTGGTGG-3′
NK_attP1-FP-DXS	5′-GGGGACAAGTTTGTACAAAAAAGCAGGCTTAATGGCTTCTTCTGCATTT-3′
NK_attP4-RP-DXS	5′-GGGGACAACTTTGTATAGAAAAGTTGGGTGAAACAGAGCTTCCCTTGG-3′
NK_attP1-FP-DXR	5′-GGGGACAAGTTTGTACAAAAAAGCAGGCTTAATGACATTAAACTCACTA-3′
NK_attP4-RP-DXR	5′-GGGGACAACTTTGTATAGAAAAGTTGGGTGTGCATGAACTGGCCTAGC-3′

Table S2. Vectors used for the construction of the different plant expression vectors.

pENTR™/D-TOPO®	Invitrogen Thermo Fisher Scientific (Carlsbad, USA)
pUBQ10-Dest	[1]
pMDC107	[2]
pH7FWG2,0-Dest	[3]
pB7RWG2,0-Dest	[3]
pFRETgc-2in1-CC	[4]
pBiFCt-2in1-CC	[5]
pDONR221-P1P4	Invitrogen Thermo Fisher Scientific (Carlsbad, USA)
pDONR221-P3P2	Invitrogen Thermo Fisher Scientific (Carlsbad, USA)
pENTR-L1-GentR-L4	[5]
pB7FWG2,0-DXS	Gift from Manuel Rodriguez-Concepcion; [6]
pB7FWG2,0-DXR	Gift from Manuel Rodriguez-Concepcion; [6]
CD3-999 pt-rk (Plastids, mCherry)	[7]

References:

1. Wanke, D.; Hohenstatt, M.L.; Dynowski, M.; Bloss, U.; Hecker, A.; Elgass, K.; Hummel, S.; Hahn, A.; Caesar, K.; Schleifenbaum, F.; Harter, K.; Berendzen, K.W. Alanine Zipper-Like Coiled-Coil Domains Are Necessary for Homotypic Dimerization of Plant GAGA-Factors in the Nucleus and Nucleolus. *PLoS ONE* **2011**, *6*, e16070.
2. Curtis, M.D.; Grossniklaus, U., A Gateway Cloning Vector Set for High-Throughput Functional Analysis of Genes in Planta. *Plant Physiol.* **2003**, *133*, 462–469.
3. Karimi, M.; Inzé, D.; Depicker, A. GATEWAY™ vectors for Agrobacterium-mediated plant transformation. *Trends Plant Sci.* **2002**, *7*, 193–195.
4. Hecker, A.; Wallmeroth, N.; Peter, S.; Blatt, M.R.; Harter, K.; Grefen, C. Binary 2in1 Vectors Improve in Planta (Co)localization and Dynamic Protein Interaction Studies. *Plant Physiol.* **2015**, *168*, 776–787.
5. Grefen, C.; Blatt, M.R. A 2in1 cloning system enables ratiometric bimolecular fluorescence complementation (rBiFC). *Biotechniques* **2012**, *53*, 311–314.
6. Perello, C.; Llamas, E.; Burlat, V.; Ortiz-Alcaide, M.; Phillips, M.A.; Pulido, P.; Rodriguez-Concepcion, M., Differential Subplastidial Localization and Turnover of Enzymes Involved in Isoprenoid Biosynthesis in Chloroplasts. *PLoS ONE* **2016**, *11*, e0150539.
7. Nelson, B.K.; Cai, X.; Nebenfuhr, A. A multicolored set of in vivo organelle markers for co-localization studies in Arabidopsis and other plants. *Plant J.* **2007**, *51*, 1126–1136.
